# Psychiatric manifestations of Wilson’s disease

**DOI:** 10.1192/j.eurpsy.2021.668

**Published:** 2021-08-13

**Authors:** R. Mota Freitas, M.T. Valadas

**Affiliations:** 1 Departamento De Psiquiatria E Saúde Mental, Hospital do Espírito Santo de Évora, Évora, Portugal; 2 Serviço De Psiquiatria, Unidade Local de Saúde do Baixo Alentejo, Beja, Portugal

**Keywords:** neuropsychiatry, Wilson’s Disease

## Abstract

**Introduction:**

Wilson’s Disease is a rare, autosomal recessive disorder related to disturbances of copper metabolism. Its clinical picture includes hepatic, neurologic, psychiatric, and systemic manifestations. Psychiatric symptoms are frequent over the course of this disease and can be found in up to a quarter of patients at presentation. Successful treatment for Wilson’s Disease can be achieved using anti-copper agents.

**Objectives:**

We aim to review the literature regarding the psychiatric manifestations of Wilson’s Disease. We also include brief considerations about their management.

**Methods:**

We performed an updated review in the PubMed database using the terms “Wilson’s Disease” and “Psychiatric manifestations”. The included articles were selected by title and abstract.

**Results:**

Psychiatric manifestations, including psychosis, mood disorders, personality disorders and cognitive impairment are common in Wilson’s Disease and can be the initial symptoms of this condition. The diagnosis of Wilson’s Disease in people presenting with psychiatric symptoms heralds special considerations in psychopharmacology since this population has a higher risk of hepatic impairment, epilepsy, and extrapyramidal side effects.

**Conclusions:**

Psychiatric symptoms are common in Wilson’s Disease and can be its presenting clinical features. Missing the diagnosis of Wilson’s Disease can stall an efficient treatment and lead to inadequate patient management.

